# Alginates Combined with Natural Polymers as Valuable Drug Delivery Platforms

**DOI:** 10.3390/md21010011

**Published:** 2022-12-23

**Authors:** Katarzyna Kruk, Katarzyna Winnicka

**Affiliations:** Department of Pharmaceutical Technology, Medical University of Białystok, Mickiewicza 2c, 15-222 Białystok, Poland

**Keywords:** alginate, polymer blends, biomedical applications, drug delivery platforms, natural polymers

## Abstract

Alginates (ALG) have been used in biomedical and pharmaceutical technologies for decades. ALG are natural polymers occurring in brown algae and feature multiple advantages, including biocompatibility, low toxicity and mucoadhesiveness. Moreover, ALG demonstrate biological activities per se, including anti-hyperlipidemic, antimicrobial, anti-reflux, immunomodulatory or anti-inflammatory activities. ALG are characterized by gelling ability, one of the most frequently utilized properties in the drug form design. ALG have numerous applications in pharmaceutical technology that include micro- and nanoparticles, tablets, mucoadhesive dosage forms, wound dressings and films. However, there are some shortcomings, which impede the development of modified-release dosage forms or formulations with adequate mechanical strength based on pure ALG. Other natural polymers combined with ALG create great potential as drug carriers, improving limitations of ALG matrices. Therefore, in this paper, ALG blends with pectins, chitosan, gelatin, and carrageenans were critically reviewed.

## 1. Introduction

Beneficial properties of alginates (ALG) enable their wide application in the pharmaceutical, medical, and food industries [1]. ALG were discovered at the end of the nineteenth, century and their commercial production started in the United States in 1929. In 1983, the Food and Drug Administration (FDA) approved the use of ALG as a food ingredient [2]. Since then, ALG have been investigated thoroughly and have been the subject of many studies. ALG are polymers of natural origin, and their main source of acquisition is brown algae (*Phaeophyta*). They occur most frequently in algae cell walls [2]; however, they can be found in the intracellular matrix in *Laminaria* and *Fucus* species [3]. The most common form of ALG, which appears in cell walls, is calcium salt [4], with sodium or potassium salts appearing less frequently. The algae species most commonly used for ALG acquisition are inter alia: *Ascophyllun, Macrocystis, Laminaria, Eisenia, Ecklonia, Fucus, Alario, Necrocystis, Sargassum, Ascophyllum nodosum, Macrocystis porifera*, and *Laminaria* [1,2,5,6]. Another valid means of obtaining ALG is technology using bacteria from *Pseudomonas sp*. or *Azotobacter (Acetobacter) sp*. However, this technology is mostly used on a laboratory scale due to the low economy of the process [6]. An undeniable advantage of using bacteria in the process of obtaining ALG is the possibility to design the desired ALG structure.

## 2. The Chemical Structure and Physical Properties of ALG

“Alginates” is an umbrella term encompassing various different chemical compounds. The basic one is alginic acid (AA), which occurs as sodium, potassium, magnesium, calcium, or ammonium salts. It can also build salts with other elements such as strontium, barium, and lead. A synthetic ALG derivative is propylene glycol alginate.

Depending on the derivative, the properties of ALG can be different (Table 1). AA is described as a white to yellowish fibrous powder without any smell or taste. It is freely soluble in alkali metal hydroxides and practically insoluble in 95% ethanol or other organic solvents. AA swells but does not dissolve in water. It can bind up to 300 times more water than it weighs [7]. Sodium alginate (SA) occurs as a tasteless and odorless white to pale yellow-brown powder. It has good solubility in water and forms viscous colloidal solutions. The substance can increase its volume by up to 10 times [5]. The viscosity of SA aqueous solutions depends on several variables, such as the degree of polymerization, pH, presence of ions, length of the chain and the polymer concentration. Furthermore, 1% SA solution, depending on molecular weight, has a viscosity between 20 and 400 mPa·s at a temperature of 20 °C. The viscosity decreases with the temperature increase and pH increase above 10 [5,8]. SA water solubility increases with increasing guluronic acid content in the polymer chain [6]. SA of bacterial origin does not show such a dependence [6]. SA is poorly soluble in 95% ethanol, ether, chloroform, water and ethanol mixes and water at pH < 3 [5,8]. Calcium alginate (CA) is similarly described as a tasteless and odorless white to yellow-brown powder or fiber. It dissolves in dilute solutions of sodium citrate, chloride and bicarbonate and in alkaline solutions, and is practically insoluble in water and organic solvents such as ether, chloroform, and ethanol [9]. Ammonium alginate occurs as white to yellow-brown threaded, granular or powdered forms, soluble in water and practically insoluble in both ether and ethanol [10].

**Figure 1:**
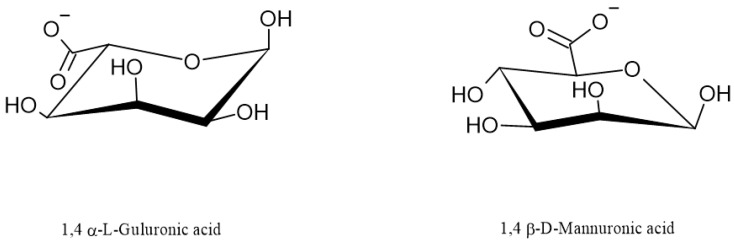
Schematic drawing of guluronic and mannuronic acid residues.

**Figure 2:**
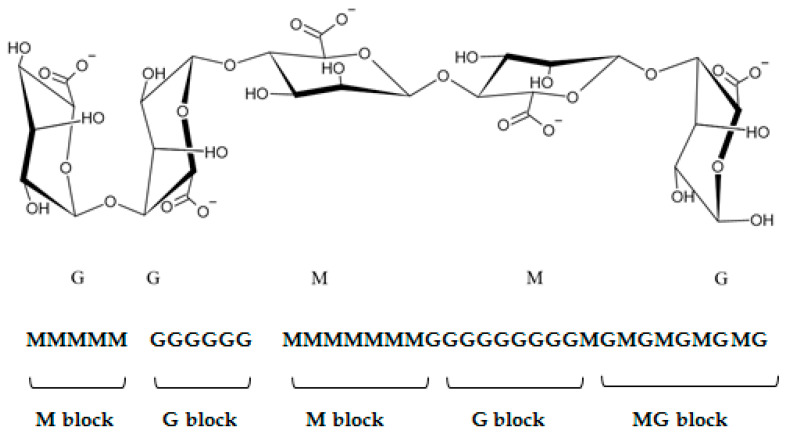
The structure of ALG chain and blocks arrangement.

Regarding their chemical structure, ALG are hydrophilic anionic polymers consisting of a polyuronic acid mixture. As mentioned above, the term ALG usually refers to AA or its salts, but it can also be used for other ALG derivatives such as propylene glycol esters. All ALG are linear biopolymers composed of 1,4-linked-β-D mannuronic acid residues (M—residues) and 1,4,-linked-α-L-guluronic acid residues (G-residues), which are C5-epimers of β-D-mannuronic acid (Figure 1) [2]. The residues can be arranged differently in the polymer chain; they can alternate and form heterogeneous structures (for example MGMGMGGM) or homogenous structures called blocks: M-block (sequence poly M) and G-block (sequence poly G) [1,7,12]. M-blocks and G-blocks are scattered throughout the polymer chain between the sequences of altering M- and G-residues (Figure 2) [2]. The chemical structure of ALG determines their physical characteristics, which are frequently influenced by the source of acquisition [2]. Mannuronic acid residues are dominant in young algal plethora. They can be caused by the epimerization of mannuronic acid to guluronic acid undertaken by the enzyme C5-epimerase as the algae age. In addition, the content of both acid residues differs depending on the harvest season of some algae species. ALG produced by bacteria have an additional acetyl group in both the mannuronic acid and guluronic acid residues [6,13].

The chemical and physical properties of ALG are determined by the chemical structure of the polymer chain, its length, and consequently its molar mass, sequence of M- and G-residues, the length of each sequence, glycosidic bonds configuration [5], and the content of each acid residue in the whole polymer mass. Approximately 200 commercial ALG varieties are available [14], with a molar mass within the range of 33,000–400,000 g/mol. The content of M- and G-residues and their arrangements in the polymer chain have the strongest influence on the physical and chemical properties of ALG. Gelling properties are stronger for ALG rich in G-residues in comparison to those rich in M-residues. Gels formed by ALG rich in G-residues are stiffer, harder, more brittle and fibrous, and the gelling process is slower compared to gels formed by the ALG rich in M-residues [1,15]. Because ALG of bacterial origin contain more M-residues, they form more elastic and less viscous gels [13,16]. Due to the limited rotation around the 1,4-glycosidic bonds, the ALG chain is a stiff structure. Moreover, sugar residues are rigid and negatively charged, which contribute to the chain stiffness. The more G-residues in the chain, the more rigid the ALG chain is [17]. The polymer chain stiffness increases in order to block the content MG<MM<GG [17]. MG-blocks determine the shrinkage and flexibility of ALG gel. Due to this fact, ALG rich in M-residues absorb more water compared to ALG rich in G-residues. As a result, ALG with prevalent M-blocks can exchange ions with the environment more easily. This ability is used in ALG dressings formulation [18,19,20,21]. Calcium ions from CA present in the dressing are exchanged with sodium ions from the wound. Consequently, CA in the dressing transforms into SA, which dissolves in water and creates a gel, hydrating the wound and, as a result, accelerating and facilitating the healing process. The gel loses its integrity and stability due to the release of ions, but on the other hand, the released calcium ions induce the coagulation process in wounds [22].

### The Gelling Properties of ALG

**Figure 3:**
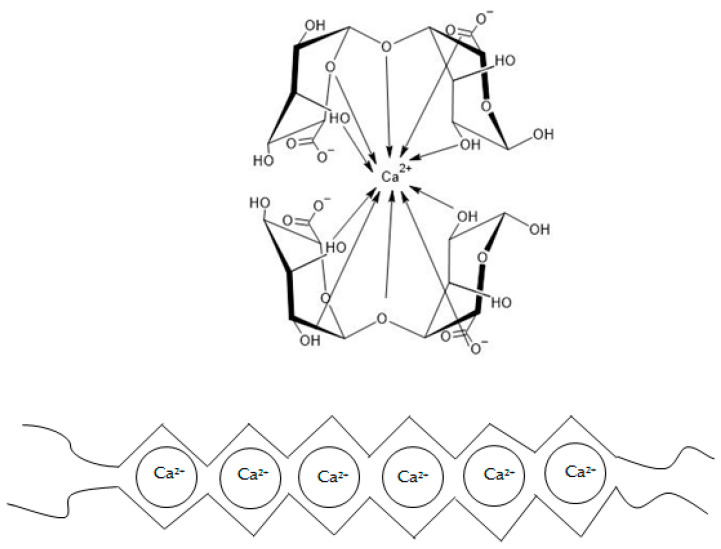
The “egg-box model” of ALG ionic gelation (modified, according to [25]).

One of the most commonly utilized properties of ALG is their ability to form gel systems. The gelation mechanism strictly depends on the repulsive force of negatively charged sugar residues and on the ratio and distribution of M- and G-residues in the polymer chain [17]. There are several theories about the mechanism of ALG gelation. Generally, gels are formed in the presence of several divalent ions such as calcium, strontium, barium, cadmium, cobalt, copper, manganese, nickel, lead, and zinc, or trivalent ions such as ferrous and aluminum [2]. Magnesium ions do not induce the process of gel forming [6]. The most commonly referenced gelation mechanism is called the “egg-box model” (Figure 3). It was confirmed that G-blocks have a higher affinity for calcium ions in comparison to M- or MG-blocks [17]. Guluronic acid residues can bind covalently to calcium ions. In other words, calcium ions fit into negatively charged niches formed by G-blocks in the polymer chain like eggs in an egg carton [23,24]. Therefore, the higher the G-blocks content, the more rigid/brittle and stronger the gels are formed [17]. On the other hand, M-blocks create flatter structures, which cannot provide enough space to bind calcium ions. As a consequence, the affinity of M-blocks for calcium is lower compared to G-blocks. In the presence of calcium ions, the M-blocks act as a factor that makes the ALG gel structure more flexible. The brittle and rigid gel created in the G-block area is interspersed and shuffled with the looser one, formed by the M- or MG-blocks [17]. Lanthanum, praseodymium, and neodymium ions have an affinity for both G- and M-blocks.

ALG gelation can also be induced by decreasing the pH below the ALG monomers’ pKa through the addition of lactones such as d–glucono–δ–lactone [1,2,8].

Calcium-ion-induced gelation is a fast process, which is difficult to control and terminate. The gelling speed is a relevant factor because controlled gelling enables obtaining a homogenous gel structure and its mechanical integrity [25,26]. It is possible to control the process or even reverse it by adding citrate ions or phosphate buffer (pH 7), which competes with carboxyl residues from ALG for calcium ion binding [6]. The ALG gelation process is more controllable when using gluconolactone [22], glacial acetic acid, calcium sulfate, or calcium carbonate as crosslinking factors. To better control the gelling process, a lower temperature [27] or freeze–thaw technology [28] can be utilized.

## 3. Biological Properties of ALG

Due to the low toxicity, ALG are considered by the FDA to be generally recognized as safe (GRAS) [29] and are included in the Inactive Ingredient Database [30]. In Europe, ALG can be non-parenteral drug excipients/ingredients. Canada has placed ALG on the List of Acceptable Non-medicinal Ingredients [8]. ALG non-immunogenicity, non-toxicity, and biocompatibility after nasal [31], ocular [32], and oral [33] administration were confirmed. 

ALG possess biological activity per se (Table 2). They create a floating gel structure, which due to its mucoadhesive properties, remains on the gastric mucosa surface for a fairly long time, protecting it from irritation [2,5]; therefore, ALG are exploited as a physical barrier in reflux disease or heartburn. ALG have been proven to suppress postprandial insulin secretion by modulating gastric emptying or reducing intestinal glucose uptake through the inhibition of glucose transporters [34,35,36], which could be related to α-amylase activity reduction as well [37,38,39]. Moreover, ALG are characterized by an anti-hyperlipidemic effect due to their ability to bind bile acids in the gastrointestinal tract and reducing absorption. In the rat model, CA showed a reduction in the amount of cholesterol in the cytoplasm after oral administration [40]. 

ALG are also characterized by antimicrobial activity [41,42]. It was shown that negatively charged ALG molecules can react with the bacterial cell wall, leading to its destabilization and leakage of intracellular fluids [43,44]. There are also theories stating that ALG gel surrounding the microbial cell inhibits the influx of nutrients into the cell [43]. ALG antibacterial activity has been described for many bacteria species such as *Pseudomonas* spp., *Escherichia* spp., *Proteus* spp. and *Acinetobacter* spp. [45,46]. Antiviral activity is observed for ALG-sulfated derivatives because of their stronger negative charge. ALG molecules protect host cells from contact with viruses. The antiviral effect of ALG might also be related to their immunomodulatory properties through activation of macrophage cytokine secretion. Antiviral activity has been investigated for viruses of *Flaviviridae, Togaviridae, Rhabdoviridae* and *Herpesviridae* families [42,47,48,49,50,51].

Since ALG are polysaccharides, they might also exhibit immunomodulating properties. Polysaccharides possess the ability to stimulate a host immune response through activation of antigen-recognizing cells. Studies on fish proved the immunomodulatory properties of ALG. It was shown that Ergosan, a seaweed extract rich in AA, stimulated lysozyme secretion in sturgeon *Huso huso* L. [52], and AA stimulated innate immunity in sea bass *Dicentrarchus labrax* L. [53]. AA supplied with food at a level of 0.4% strengthened the immune system of *Cirrhinus mrigala* [54].

ALG are also tested for their anti-inflammatory effect, since most pathological conditions are characterized by the development of inflammation. It is postulated that ALG could be an inhibiting factor for the inflammatory process on the mucous membrane of the esophagus or mouth caused by radiation stomatitis [3]. Studies on rats with induced arthritis showed positive effects of AA on the reduction of swelling and erythema in the course of joint inflammation [55,56]. Using HaCaT lineage keratinocytes, it was found that AA reduces oxidative stress, inhibits macrophage activation, and decreases the activity of myeloperoxidase (MPO) [57]. To date, a number of studies have indicated a potential tendency for ALG to exhibit anti-anaphylactic properties [5]. It is postulated that it might be due to the inhibition of histamine secretion from mast cells [58]. The following negative feedback results in inhibition of histidine decarboxylase and expression of proinflammatory cytokines. This study demonstrated a reduction in histamine release from rat peritoneal mast cells after administration of AA. Moreover, a reduction in IgE production in the serum of mice immunized with β-lactoglobulin after administration of AA was reported [59].

It appears that ALG can activate blood coagulation through the thrombin activation pathway. In addition, ALG are thought to induce TNF production and, by activating blood leukocytes, facilitate wound healing [6]. Moreover, potassium alginate (PA) could prevent complications of hypertension, such as myocardial and renal hypertrophy and the occurrence of stroke [60]. There are also some studies on their application in the treatment of obesity and diabetes [1].

**Table 2:** ALG biological properties.

ALG Biological Properties	Reference
Promote coagulation	[1,6]
Antacid	[2,5]
Suppressing postprandial insulin secretion	[34,35,36]
Antibacterial	[45,46]
Antiviral	[42,47,48,49,50,51]
Immunomodulatory	[52,53,54]
Anti-inflammatory	[3,55,56,57]

## 4. Pharmaceutical Applications of ALG

Among many advantages of ALG, SA monographs are included in both European Pharmacopeia and United States Pharmacopeia [61,62]; thus, their properties for pharmaceutical and biomedical use are strictly regulated. Due to the low toxicity, biocompatibility and inert character, European Food Safety Authority (EFSA) approved ALG for use in a wide range of products, even in young children with special delivery requirements. The versality of ALG leads them to be widely applied in drug delivery systems, such as micro-, nanoparticles, tablets, semi-solid drug forms (as thickener and suspending agent), in tissue engineering, bone and cartilage regeneration, and in wound treatment (ALG dressings). Drug dosage forms designed with the use of ALG are mainly micro-, nanoparticles, tablets, capsules, hydrogels, and beads [63,64].

**Figure 4:**
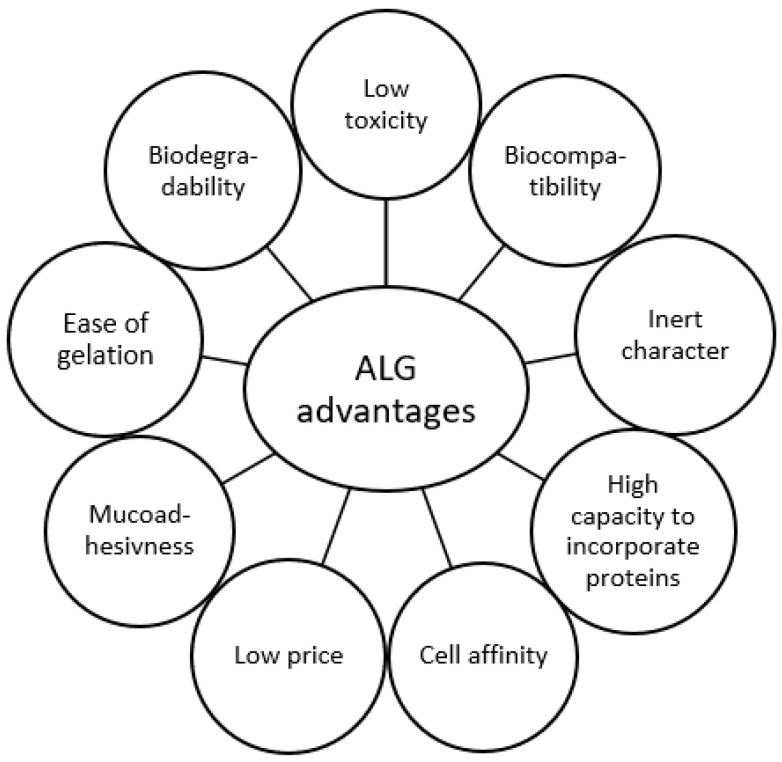
Advantages of ALG as pharmaceutical excipient.

Such a wide application of ALG is also possible due to other numerous advantages, such as gelation properties, cell affinity, and high capacity to incorporate and release various active substances, including proteins (Figure 4) [63]. Moreover, ALG are natural and relatively inexpensive polymers. Mucoadhesiveness of ALG makes them a promising excipient for mucosal (ocular [65], nasal [66], vaginal [67], oral [68]) delivery systems.

### 4.1. Microparticles

ALG microparticles are being studied to protect the drug from degradation as well as modify, release, and increase drug bioavailability. Drugs encapsulated in microparticles can be both hydrophilic and hydrophobic. Due to biocompatibility, biodegradability, non-toxicity, relatively low cost, properties favorable for the spray-drying process, and ease of gelation, SA presents itself as an excellent matrix material for microparticles technology [15,69,70,71]. ALG microparticles are biocompatible and highly bioavailable dosage forms [72], they are relatively small (1–500 μm), possess a large surface area, protect the core from external agents, mask taste and odor, and enable different release modification [73,74,75,76]. Microparticles are also delivery platforms for proteins, nucleic acids, enzymes, cells, growth factors and genes in tissue engineering [77,78,79,80]. However, disadvantages of microparticles include relative high production costs, specialized equipment, low reproducibility of the process and instability of the final product [81]. External gelation is one of the most often used methods to obtain microparticles by dropping ALG solution into the crosslinking medium [13]. Particle size is a key parameter in the context of drug distribution in the gastrointestinal tract, drug release and degradation process [15]. Currently, the tendency is to receive the finest possible microparticle size by enhancing the dripping method through the involvement of external forces, such as vibration, electrostatic force, or coaxial air flow. It is also possible to improve the emulsification method by using sonification, membrane emulsification, or microfluidic methods [82,83]. Microcapsules obtained by external gelation provide a protective coating for sensible components such as living cells, cytokines, growth factors and proteins [84,85,86,87,88]. ALG microparticles are semi-permeable and provide immune protection to cells, which allows them to differentiate and proliferate [89]. Martin et al. [90] proposed a novel mucoadhesive system consisting of ALG microparticles loaded with nystatin for the treatment of oral candidiasis to increase residence time on the buccal mucosa and to improve the effectiveness of the treatment. Designed microparticles demonstrated antifungal activity for up to 48 h in an in vitro study. An interesting approach to the topic was demonstrated by Benavides et al. [91], Faidi et al. [92] and Fermandizz et al. [93], who proposed thyme, clove essential and cedar wood oil-loaded ALG microparticles to protect against evaporation and disintegration under the influence of oxygen, UV, and heat. All the researchers obtained microparticles with relatively high encapsulation efficiency, however further studies are required to refine the conditions for microparticle production. Hussein N. et al. [94] designed ALG microparticles loaded with ropinirole hydrochloride using the spray-drying method for intranasal delivery. Ropinirole is an agonist of D_2_ dopamine receptors in the brain, employed in the treatment of Parkinson’s disease in monotherapy or in combination with other drugs, most frequently levodopa. The drug possesses an approximately 50% hepatic first-pass effect; thus, it is characterized by low bioavailability. ALG microparticles were utilized in this study as a drug form for intranasal use to increase ropinirole bioavailability by reducing the first-pass effect, since the nasal mucosa possesses a relatively large absorption area (approx. 150 cm2), and the drug reaches its target site of action faster: the nervous system. In addition, ALG microparticles, due to their mucoadhesive properties, prolonged the contact of the formulation with the mucosa. The study showed that designed particles were non-toxic to an isolated sheep mucosa and were stable for 2 months of storage at 5 °C ± 1 and 20 °C ± 2. There have also been attempts to load insulin into ALG microparticles [95,96,97,98]. To improve the comfort of insulin administration, novel drug delivery systems, including ALG microparticles, are being developed to protect insulin from degradation. Mild conditions for producing microparticles by the internal gelation method did not damage its secondary structure [96], and microparticles prepared from SA blended with mucin showed a hypoglycemic effect in diabetic rats comparable to subcutaneous administration of insulin [97]. Insulin loaded into microparticles by the spray drying maintained its bioactivity [98].

ALG microparticles were also utilized to improve the stability of papain during storage and enteral release, which extended the shelf life of papain during storage to about 3.6 years, compared to free papain (about 0.48 years) and a higher rate of drug release at pH >6.8 than at pH <5 [99]. A similar premise supported the design of hydrogel beads with subtilisin on a matrix of SA blended with guar gum [100] or the encapsulation of plasmid DNA (pDNA) encoding a green fluorescent protein (GFP)-reported gene using SA [101]. Szekalska M. et al. [102] designed ALG microspheres with ranitidine hydrochloride to improve their bioavailability after oral administration, and the obtained formulations were characterized by sustained drug release and beneficial mucoadhesive properties.

Microparticles can also provide protection for orally administered probiotic bacteria from harmful environmental factors, such as stomach acidic pH, and high temperature during the manufacturing and storage process. Faarez I.M. et al. [103] placed *Lactobacillus plantarum* lactic acid bacteria (LAB)12 cells in ALG microcapsules encapsulated with cellulose derivatives: methylcellulose (MC), sodium carboxymethylcellulose (NaCMC) or hydroxypropyl methylcellulose (HPMC) to increase heat resistance. Bacteria cells gained higher survivability during storage when HPMC or MC were blended with ALG. Mirmazloum I. et al. [104] encapsulated *Lactobacillus acidophilus* with Reishi medicinal mushroom (*Ganoderma lingzhi*) extract as a prebiotic for the bacteria in a matrix composed of SA blended with maltose, HPMC or hydroxyethyl cellulose (HEC) to increase the stability of the formulation and to mask the bitter taste of the fungus, which resulted in increased stability of the formulation during storage and slower release, especially with the addition of maltose.

### 4.2. Nanoparticles

Nanoparticles are characterized by the same order of size as proteins and by a relatively large surface area, which creates the possibility of placing specific ligands [105,106,107]. In addition, nanoparticles enable modifications in drug pharmacokinetics, they can reduce drug toxicity and the possibility of damaging healthy cells, and they enable precise drug delivery to the targeted site of action, especially in targeted cancer therapy [107]. Nanoparticles can also improve solubility and bioavailability of poorly water-soluble substances and can provide modified drug release while reducing toxicity [15,64]. Moreover, nanoparticle size enables passage through the smallest capillaries [15], and they are characterized by the ability to inhibit P-glycoprotein activity, thus to reduce the resistance of tumor cells to cytostatics [108]. In order to improve the bioavailability of metformin, to reduce its side effects and to obtain sustained drug release, Kumar S. et al. [109] developed metformin-loaded ALG nanoparticles. The study conducted on adult Wistar albino rats showed sustained drug release from nanoparticles (up to 30 h), and a lower dose of the drug was needed. Thomas D. et al. [110] proposed ALG nanoparticles loaded with rifampicin to improve drug bioavailability after oral administration and to obtain controlled drug release. The study showed a pH-dependent release profile with controlled release of the drug for 6 h at pH 7.4. Ahmad Z. et al. [111] developed ALG nanoparticles with isoniazid, rifampicin, pyrazinamide or ethambutol as an oral delivery system for tuberculosis treatment to improve pharmacokinetics of these drugs and to reduce the high potential of side effects. The researchers created nanoparticles with high encapsulation efficiency and higher bioavailability of encapsulated drugs compared to free drugs. Higher tissue concentrations of drugs were observed on a Laca mice model after administration in encapsulated form compared to free drugs. In addition, tissue concentrations were maintained at >MIC levels for 15 days, making it possible to use these drugs less frequently than daily. Kirtane A.R. et al. [68] developed ALG nanoparticles for a chemiotherapeutic drug, doxorubicin, in order to improve its oral bioavailability. A study on Madin–Darby Canine Kidney II cells (MDCK) showed improved doxorubicin transport across tumor cells overexpressing P-glycoprotein after nanoparticle administration. Studies on a mice model showed higher oral drug bioavailability during nanoparticle administration compared to free drugs. Bakhshi M. et al. [112] designed a vaccine against *Escherichia coli* 0157:H7 consisting of IgY encapsulated within ALG nanoparticles. The study showed that IgY was released from the nanoparticles in the stomach in a minimal amount (up to 10%); thus, the formulation managed to protect the drug against low pH and did not adversely affect the biological activity of immunoglobulins.

### 4.3. Tablet Technology

ALG are used in a variety of applications in tablet technology. SA at a concentration of 1–5% acts as a disintegrating agent in powder form and in the form of a 1% solution as a binder. ALG are also used as fillers as well as taste and odor maskers. Controlled drug release might be achieved through the application of ALG matrix tablets, which undergo superficial swelling and slow dissolution. Thus, drugs that are well soluble in water slowly dissolve in water, flowing into the tablet interior, and such a solution diffuses to the outside of the tablet. In contrast, substances that hardly dissolve in water are released from the matrix by erosion of the tablet as it passes through the gastrointestinal tract [12]. The biopharmaceutical properties of ALG tablets significantly depend on intrinsic factors, namely the properties of the ALG itself. The molecular weight directly proportionally affects the viscosity of the ALG solution, and thus, an increase in viscosity entails a slower release from the matrix tablet [113]. The concentration of ALG in the tablet affects the release rate in a similar way [114]. The M/G ratio is also not without influence, ALG with higher G-content form stiffer gel structures, which slow down drug release [13,115].

## 5. Biomedical Applications of ALG

### 5.1. Tissue Regeneration

The application of ALG in tissue engineering and bone and cartilage regeneration seems interesting and potentially promising, as treating damage in these tissues is often a difficult and lengthy process [22]. The most desirable strategy is to stimulate osteogenesis and chondrogenesis in situ [91], which can be achieved by supplying the site of damage with stem cells capable of proliferation and differentiation [91,116,117,118,119,120,121]. As ALG gels are characterized by a structure similar to the extracellular matrix in tissues, they are being studied for potential use in tissue engineering or cell transplantation [22]. The principle of gel action is to deliver cells to a specific location in the body and to provide conditions for tissue reconstruction [122,123]. The influence of cells such as osteoblasts, chondrocytes, or bone marrow mesenchymal cells (MCSs) on osteogenesis and chondrogenesis is being studied [116,124,125,126,127]. Numerous studies showed bone regeneration using injectable ALG scaffolds containing MCSs [120,121,124,125,126,127,128,129]. ALG can be inserted into tissues in a non-invasive manner, they fill irregular spaces accurately, are easily chemically modified, and possess good regenerative properties, as proven in an animal model [118,130]. In mouse embryonic stem cell studies, the ability of ALG to promote stem cell differentiation into bone cells was demonstrated [131]. ALG gels are biodegradable and do not possess sufficient mechanical properties to allow for load transfer during the initial stages of regeneration [22]. In order to improve mechanical properties, ALG were mixed with ceramides, hydroxyapatite [132,133], CH [134], or bio-glass [135]. ALG gels might also be carriers for growth factors such as bone morphogenetic proteins (BMPs) [83] or tumor growth factor β (TNF-β) [124,125,126,128].

### 5.2. Wound Care

One of the most common dressings used for centuries has been gauze, which is easy to use, inexpensive, and has a high water absorption capacity. However, it can stick to the wound and cause re-damage during dressing changes [91,136]. Current emphasis is on modern dressings providing a moist wound environment while managing the exudate. Among many advantages of ALG dressings are biocompatibility, optimal water vapor permeability, mild antiseptic properties combined with non-toxicity, and biodegradability [91,137]. The principle of ALG dressings is to absorb exudate from the wound, exchange calcium ions from the dressing for sodium ions from the wound, convert to gel form and provide moisture to the wound. These processes promote granulation and epithelialization of the epidermis and thus wound healing [22,138,139]. The ratio of mannuronic to guluronic residues affects the ability to absorb exudate [140,141]. A high content of mannuronic acid is positively correlated with the ability to retain water; however, the fibers of such dressings are weaker. Some studies have indicated that pathogens from the wound were trapped in the gelled structure of the dressing [142]. The manufacturing process of ALG dressings begins with crosslinking the ALG with calcium ions and impregnating the material with the resulting gel. Such a semi-finished product is freeze-dried and mechanically smoothed to obtain flexible, delicate fiber mats [143] or foam sheets [144]. To improve the properties of ALG dressings, several compounds such as silver [145], zinc ions [146,147,148,149,150,151,152,153,154], chitosan (CH), fucoidan, asiaticoside [155], gelatin (GEL) [91], polyvinyl alcohol (PVA) [156,157], or cellulose [158] can be introduced. The aim of adding these components is to improve antibacterial properties (silver, zinc ions, CH), swelling rate, tensile strength (PVA), and other mechanical properties (fucoidan, GEL, cellulose). Another excipient added to ALG dressings are antibiotics, such as clindamycin [157] vancomycin [159], aminoglycosides [160], curcumin [149], aloe vera [154], or active carbon [161] for the elimination of unpleasant wound odor. An interesting approach is to enrich ALG dressings with oxygen release microspheres (ORMs) in order to provide oxygen and thus to facilitate neovascularization and cells proliferation and to promote wound healing (Table 3) [162].

## 6. Utilization of ALG Mixed with Selected Naturally Derived Polymers

**Figure 5:**
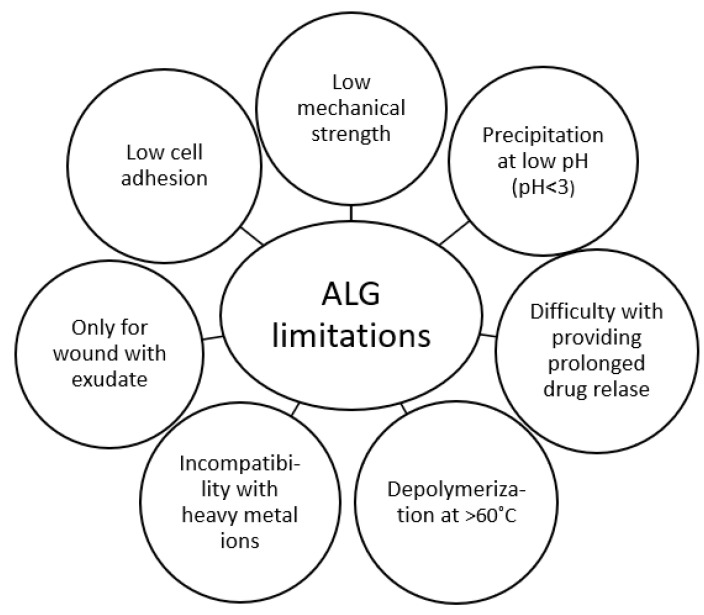
ALG limitations [63].

As a result of numerous advantages, ALG are widespread polymers in biomedical technology. Nevertheless, ALG possess certain limitations in use, such as low mechanical strength, incompatibility with heavy metal ions, depolymerization at temperatures >60 °C, and consequently reduced viscosity and precipitation at low pH (Figure 5) [63]. In this work, blends of ALG with selected natural polymers were described to ameliorate the aforementioned drawbacks (Table 4).

### 6.1. ALG Blends with Pectins

**Figure 6:**
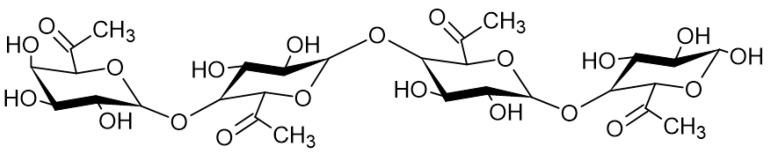
Homogalacturonan structure.

Pectins (PEC) are natural heteropolysaccharides found in the cell walls of higher plants [163] composed of mainly (1→4)-α-D-galacturonic acid residues and α-1,2- rhamnose, which form segments consisting of homogalacturonan (Figure 6) (HG), rhamnogalacturonan-I (RG-I), rhamnogalacturonan-II (RG-II), xylogalacturonan (XGA), arabinan, arabinogalacturonan I, and arabinogalacturonan II [164,165].

The applications of PEC in both food and pharmaceutical industries are mainly related to their ability to form a gel structure [166,167]. The structure of PEC might be modified by esterification, amidation, etherification, or oxidation, which affect the gelation properties of PEC. PEC themselves have been shown to possess hypoglycemic [168,169,170], cholesterol-lowering [171,172], and antiproliferative effects in prostate, colon, or pancreatic cancers [173,174], and to provide a medium for probiotic bacteria residing in the colon [175]. Due to their advantages, such as the ability to gel in acidic environments, microbial degradability, biocompatibility, and wide possibilities of chemical modification, PEC are currently intensively studied. Madziva H. et al. [176] designed microcapsules consisting of ALG and PEC as a potential carrier for folic acid. They used SA with a viscosity of 2% solution of 250 mPa·s and a potassium salt of PEC with a methylation degree of 28% using calcium chloride as a crosslinking agent. The corresponding amounts of polymers were mixed with purified water to obtain different ratios of ALG:PEC (%, *w*/*v*): 100:0, 80:20, 70:30, 60:40, in which folic acid was suspended. The mixtures were dropped into calcium chloride solution to form microcapsules, which were left in a dark place to harden. Formulations with higher ALG content (ALG100:PEC0 and ALG80:PEC20) were characterized by a regular shape, in contrast to microcapsules with higher PEC content (ALG70:PEC30 and ALG60:PEC40), which were irregular. In addition, as the PEC content of the blends increased, an increase in the encapsulation efficiency was observed (from 54.80 ± 0.2% for the ALG100:PEC0 formulation to 74.2 ± 0.92% for the ALG60:PEC40 formulation). Formulations with higher PEC content improved the retention of folic acid, which degraded relatively rapidly in ALG microcapsules (folic acid content of the different formulations was compared after 11 weeks of storage at 4 °C). Microcapsules stored in a freeze-dried form showed greater stability than air-dried microparticles. In an in vitro study at pH 1.2, significantly reduced release was demonstrated for the ALG70:PEC30 formulation compared to pure ALG, whereas at pH 8.2, folic acid release from the ALG100:PEC0 formulation was significantly faster than from the ALG70:PEC30 formulation. Pour P.K. et al. [177] designed ALG–PEC microcapsules with folic acid. They used SA with a viscosity of 2% solution 2000 mPa·s, citrus PEC, and folic acid. Microcapsules with ALG to PEC ratios of 100:0; 80:20; 70:30; 60:30 were prepared by an external gelation method by dropping folic acid in polymer solution into calcium chloride solution and then left in the darkness to harden. The encapsulation efficiency increased with the increasing PEC content in the blend up to the formulation of ALG70:PEC30. In the context of drug release in an acidic medium, the lowest release rate was observed for ALG70:PEC30 formulation, while the ALG60:PEC30 formulation had the highest release rate. Based on the SEM images, the authors observed a tendency for the spherical shape of microcapsules to disappear with increasing PEC content in the formulation. The ALG70:PEC30 formulation was found to be optimal considering all the analyzed parameters. Islan G.A. et al. [178] encapsulated ciprofloxacin into ALG- PEC gel matrices for oral administration. They used low viscosity SA and PEC with different degrees of methylation, which affected their physical properties, especially their gelling mechanism [179]: LM (low methylated) at 33%, MM (medium methylated) at 55.3% and HM (high methylated) at 74%. Microcapsules were prepared by the external gelation method by dropping the solution of antibiotic and polymer into the calcium chloride solution. The authors showed the greatest stability and encapsulation efficiency of the antibiotic at pH 4. They compared several different polymer mixtures at different concentrations and showed an increase in drug binding (from 47.2% to 71.1%) with an increase in the concentration of pure ALG (from 1% to 3%). The percentage of ciprofloxacin binding in microcapsules composed of MM and HM PEC at 1% concentration was slightly higher, compared to ALG microcapsules. The binding of the drug by LM PEC 1% was lower compared to MM and HM PEC and to pure ALG. However, mixing these polymers significantly increased the drug binding (up to 90%) compared to microparticles prepared from pure polymers. Based on the SEM images, the authors indicated a relationship between the amount of PEC in the formulation and the shape of the particles. ALG microcapsules have a spherical shape with cracks on the surface, while when blended with HM PEC, their shape was irregular, and the surface was smoother. Formulations with pure 2% ALG and 2% ALG with 1% HM PEC were chosen to evaluate the release kinetics. At pH 1.2, a much faster drug release was demonstrated for the pure ALG compared to the complex matrix, whereas under conditions simulating the environment of the small intestine (pH 7.4), the release profile of both formulations was similar, and the total percentage of ciprofloxacin released from the ALG-HM PEC matrix was about 20% lower compared to the pure ALG. Presumably, this occurred due to the interaction of HM PEC with the ALG network.

In addition to the hypolipemic effect, simvastatin has also been shown to promote wound healing by increasing angiogenesis and lymphangiogenesis, as demonstrated in a study on diabetic rats [180]. Simvastatin also possesses an anti-inflammatory effect on the skin [181], and therefore, composite polymer films composed of ALG and either PEC or gelatin (GEL) with simvastatin as an auxiliary agent were fabricated to promote the wound healing process (Rezvanian M. et al. [182]). They used SA, type B GEL (alkaline extraction) and HM PEC in the study. Films consisting only of SA at a concentration of 5%, and a mixture of SA with PEC or GEL in a weight ratio of 1:1, were prepared by the solvent casting method. The mixtures of aqueous polymer solutions with glycerol were dried at 45 °C for 48 h after being poured onto Petri dishes. The films were then incorporated with 2% ethanolic simvastatin solution and dried again. All the films obtained, both drug-containing and drug-free, were characterized by favorable mechanical properties, came off the Petri dish easily, and were smooth and flexible. The PEC-containing films were more transparent compared to the yellowish-brown GEL-containing films. The addition of PEC resulted in an increase in elongation at break, and did not significantly affect tensile strength, while GEL added to ALG, on the contrary, resulted in a decrease in tensile strength and a greater increase in elongation at break than reported for PEC. In addition, for drug-containing formulations, an increase in tensile strength and a slight decrease in elongation at break were observed compared to drug-free films, which might be related to the increase in film thickness after drug addition. The rheological properties of the films are important in the context of their use as dressings, because they absorb exudate from the wound, thus undergoing a process of rehydration return to gel form, which should be characterized by the same rheological properties that the films possessed before drying in the process of their fabrication. The viscosity of ALG and ALG–PEC gels before drying and after rehydration under conditions simulating secretion from the wound did not change significantly, in contrast to GEL-containing films, in which the rheological properties deteriorated significantly after absorbing the exudate and as a result lost the round shape given to them in the technological process. The authors also observed significant differences in film expansion during water absorption. The ALG and ALG–GEL films increased in size by about 47% and 90%, respectively, without retaining their original shape, in contrast to the ALG–PEC film, which increased in size by about 50% while retaining its shape. Thus, it was concluded that ALG–GEL dressings could be used for wounds with less exudate, while those with PEC addition might potentially handle more exudate. Films based on ALG–PEC blends were characterized by higher smoothness compared to the coarse ALG film. Moreover, only in the case of films containing ALG and PEC were distinct crystalline structures observed, which could explain the better mechanical properties of films of this composition compared to other films. The release studies showed that the highest level of simvastatin release under pH 7.4 was achieved with the ALG–GEL film, whereas the lowest was with the ALG–PEC film. The authors stated that this might be related to the expansion ratio of these films—the larger the surface area of the dressing, the higher and faster drug release. The initial rate of drug release from ALG and ALG–PEC dressings did not differ significantly; only after 18 h did the release from ALG–PEC film decrease compared to the plain ALG formulation. Drug release from the ALG–GEL dressing was faster compared to the PEC-containing formulations. For wound dressings, prolonged drug release is desirable because of the resulting reduced frequency of dressing changes. Liu C.-M. et al. [183] investigated the effect of different proportions of ALG and PEC on the properties of drug formulation. SA, HM PEC and bovine serum albumin (BSA) as model drugs were used to fabricate beads. ALG–PEC gels were made in ratios of 100:0 (ALG100:PEC0), 75:25 (ALG75:PEC25), 50:50 (ALG50:PEC50), 25:75 (ALG25:PEC75), and 0:100 (ALG0:PEC100) using calcium chloride as a crosslinking agent. Beads were then made with BSA using the internal gelation method, but the ALG0:PEC100 formulation was omitted because it failed to form a gel structure. In contrast to the previously mentioned work [182], decreased stability and gel strength with increasing PEC content in the formulation were indicated, with an increased swelling index with increasing PEC content. Further study revealed that the addition of PEC improved the encapsulation efficiency of BSA and drug loading. Considering the release assay, ALG50:PEC50 and ALG25:PEC75 formulations were characterized by lower total drug release (75.72% and 79.64%, respectively) than ALG100:PEC0 and ALG75:PEC25 formulations (87.96% and 91.19%, respectively). The researchers showed that in the simulated gastric fluid, the degree of drug release was higher with the higher PEC content. Oh G.W. et al. [184] created films consisting of a mixture of ALG and PEC at different concentrations and analyzed their physical properties. They used SA, citrus PEC with a methylation degree of ≥6.7%, and calcium chloride as a crosslinker. Films were made with the composition of ALG:PEC: 100:0; 90:10; 70:30 and 50:50. The formulation ALG90:PEC10 showed the highest tensile strength and elongation at break, suggesting that there is a point up to which PEC improved these parameters and beyond which the mechanical properties deteriorate. The swelling index test performed by immersing the films in distilled water and PBS showed the greatest swelling for formulation ALG50:PEC50 in water and for ALG100:PEC0 and ALG90:PEC10 in PBS. For all obtained films, increased swelling was observed under PBS conditions compared to distilled water. BSA was used as a model drug in the release study and was observed at 100% release from the ALG50:PEC50 formulation within 24 h and from the ALG70:PEC30 formulation within 48 h, while the ALG100:PEC0 and ALG90:PEC10 formulations released 95% of the substance after 3 days. Awasthi R. et al. [185] designed particles from a mixture of ALG with PEC and ALG with HPMC as an oral delivery of gliclazide. They used SA, low methylated PEC (LM PEC) and calcium chloride as a crosslinker. Beads were made by the external gelation method, by dropping the polymer solution with suspended gliclazide into a calcium chloride solution and subsequent washing with water and drying. Comparing the encapsulation and drug loading efficiency, better results were observed for ALG–PEC than for ALG-HPMC particles. A swelling study was also carried out at pH 1.2 and pH 5.8, which showed a higher swelling index in pH 5.8 for all formulations than in acidic pH. All tested samples showed good mucoadhesive properties, with a slight advantage in favor of blends with PEC. Gliclazide release from ALG–PEC beads was slower than from ALG-HPMC formulations in both tested pH environments. The results indicated that designed formulations could be used in gastroretentive drug form. Jelvehgari M. et al. [186] designed gastroretentive ALG–PEC particles with piroxicam as oral delivery of a colonic targeted drug. Formulations of pure ALG with increasing concentrations (ALG1, ALG2, ALG3) and mixed formulations with ALG:PEC ratios of 1:2; 2:2; 3:2; 3:1; 3:3 were manufactured. In this study, the authors used SA, PEC (degree of methylation was not reported), piroxicam, and calcium chloride as a crosslinking agent. Particles were fabricated by external gelation method, polymer solutions in appropriate concentrations were blended together, piroxicam was suspended homogeneously, and the mixture was dropped into calcium chloride solution. The resulting particles were filtered and dried. An increase in the encapsulation efficiency with increasing PEC concentration in the formulation was found as well as a decrease in this parameter with increasing ALG concentration. ALG–PEC blends showed greater mucoadhesion than formulations of pure ALG; the formulation ALG:PEC 2:2 was characterized by the highest mucoadhesion, while the formulation with ALG at the lowest concentration (ALG1) possessed the lowest mucoadhesive properties. All formulations exhibited good swelling at pH 6.8, and the highest swelling index was observed for the formulation with pure ALG at the highest concentration. Among the blends, the formulations with the most swelling were ALG:PEC 3:2 and 3:3. The gastric retention time was also investigated, which showed significantly higher retention time for the ALG–PEC blends than for pure ALG formulation. A reduction in the retention time (from 120 to 30 min) was observed with increasing ALG concentration. Among the mixed formulations, ALG:PEC 1:2 and 3:3 were characterized by the highest retention time, 480 min ± 60 and 480 min ± 120, respectively. The release study at pH 1.2 showed significantly lower release of piroxicam from the ALG–PEC formulation (1.59–3.36%) compared to pure ALG formulation (3.04–28.94%). Release from all formulations increased when the pH was changed to 6.8, and the blended formulations possessed a more prolonged release profile compared to pure ALG. In addition, an increase in the PEC content of the formulation entailed a further slowing in the release but also a decrease in the total amount of drug released at 24 h.

In order to prolong the effect of the antidiabetic repaglinide (a drug with a short half-life), its encapsulation using an ALG–PEC matrix was performed [187], and high encapsulation efficiency, improved drug bioavailability and prolonged drug release were observed. An interesting approach was the encapsulation of insulin-producing pancreatic β-cells in an ALG-based PEC bio ink with Pluronic 127 [188]. A 3D structure with desirable mechanical properties to sustain the survival of the encapsulated cells was provided.

### 6.2. ALG Blends with Chitosan

**Figure 7:**
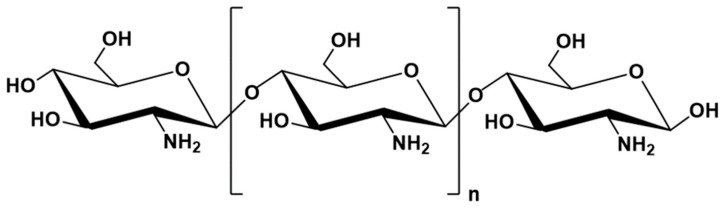
Structure of chitosan.

Chitosan (CH) is a natural cationic polymer derived by deacetylation of chitin, which belongs to the group of polysaccharides (Figure 7). Chitin ranks as the second, after cellulose [189], most abundant natural polymer in the world; it is a component of the external skeleton of crustaceans. CH is most commonly extracted from crab and shrimp carapace waste [189,190].

In terms of chemical structure, CH is a linear copolymer composed of D-glucosamine and N-acetyl-D-glucosamine resembling the structure of cellulose due to the same β-1,4-glycosidic bonds linking the sugar residues. The biological and chemical properties of CH depend on the proportion of both components in the chain. CH is a cationic polymer; thus, it possesses the ability to bind to negatively charged bacterial cell walls, which explains its antibacterial properties [191,192,193]. CH is a biodegradable and biocompatible polymer. It is characterized by mucoadhesive properties, mild gelation conditions, and ease of chemical modification, which combined with beneficial biological properties, such as the ability to chelate cholesterol, proteins and metal ions, create many opportunities for its use in biomedical and pharmaceuticals applications [194]. In order to improve antimicrobial properties of commercially available ALG dressings (Biatain), Zhao W.Y. et al. [195] enriched them with CH by immersing the dressings in an acid solution of CH. Dressings immersed in an acid solution were used as a control. The study attempted to combine the good exudate-handling properties of the ALG dressing with the antibacterial properties of CH. Based on the measurement of the inhibition zones in *Staphylococcus aureus*, and *E. coli* strains, it was shown that the ALG–CH composite dressings possessed stronger antibacterial effect, as larger zones of bacterial growth inhibition were observed. Moreover, other dressings with antibacterial activity, containing silver ions, with high antibacterial activity, were characterized by toxic effects on healthy cells (they inhibit the growth of human keratinocytes [145]), unlike the ALG–CH dressings tested. ALG interacts with CH through ionic interactions between the positively charged amino groups of the CH chain with the negatively charged carboxyl groups of the ALG. In addition, the CH molecules are characterized by the ability to chelate calcium ions from the ALG. The enrichment of the formulation with CH helped to enhance the anti-inflammatory effect of the dressing, as an immunohistochemical study showed a reduction in IL-6 levels in the wound after application of the ALG–CH dressing compared to pure ALG dressing. IL-6 is secreted by macrophages and other cells of the immune system and strongly stimulates inflammatory processes, thus reducing its level after the application of the composite dressing accelerated the wound healing process. Modulation of inflammatory processes involving faster reduction of inflammatory infiltration occurring in the wound was also demonstrated with the ALG–CH dressing, comparing it to a control sample, which was saline solution [196]. Moreover, the polyelectrolyte composite dressing possessed a stronger stimulation of fibroblast proliferation and collagen synthesis in the wound, and the scar after wound healing was less visible with the composite dressing. These results indicate that ALG and CH might complement each other in dressing technology, because dressings obtained from both polymers retained the properties of both polymers. Enrichment of ALG dressing with CH can extend its activity with an antibacterial effect and enhance its anti-inflammatory and healing effects while maintaining the properties of good exudate management, preservation of a moist environment in the wound and the effect of inhibiting bleeding. ALG in combination with CH is also being explored for use in bone repair. One of the most commonly used materials to date, hemihydrate calcium sulfate (CHS), is characterized by brittleness and rapid resorption. Encapsulation of CHS in an ALG–CH matrix was aimed at slowing down the resorption of the material by the tissues in order to prolong its presence in the bone defect, prolonging the effect of stimulating tissue regeneration and reducing the brittleness of the material to prolong the damage stabilization time [197]. In order to increase the solubility of CH in neutral pH and to facilitate the fabrication of a homogeneous polymer matrix, it was modified to form N-succinylchitosan (sCH). ALG, sCH and CHS in the form of powders were mixed with water to the consistency of thick paste, allowing for convenient application and accurate filling of the bone defect, and the addition of water resulted in the transition of calcium sulfate from the form of semihydrate to dihydrate with the release of calcium ions, which participated in the partial cross-linking of ALG, reducing the brittleness of the whole composite. Enrichment of the formulation consisting of ALG and CHS with sCH significantly improved the mechanical properties, as the values of both yield strength and Young’s modulus increased 10 times or more, which might be influenced by additional crosslinking through the interaction of -COO- groups derived from sCH with calcium ions. The formulation with CHS/ALG/sCH 50:20:30 showed the highest hardness; however, the CHS/ALG/sCH 50:30:20 formulation with slightly lower hardness showed much better application properties such as consistency, formability, and more favorable mechanical properties, which are promising for the treatment of bone defects. Considering oral drug delivery, ALG–CH combinations are being investigated to achieve controlled or prolonged drug release. Xu Y. et al. [198] designed ALG–CH particles with BSA as a model drug to provide controlled and targeted release in the small intestine. Samples with different concentrations of the two polymers were subjected to release testing under simulated gastric fluid (SGF) and then simulated intestinal fluid (SIF) conditions. In the SGF environment, an increase in drug release was observed with an increase in the CH content, which could be related to the increase in swelling degree in this environment. In the SIF environment, a gentle slowdown in the release was observed with an increase in the CH content of the formulation. The authors noted that the release profile could be further controlled if dual crosslinking was used, first with calcium ions as standard, then with sulphate ions, making it possible to obtain a highly cross-linked structure without the use of a single strong but toxic crosslinking agent such as glutaraldehyde. Thus, the designed drug formulation provided controlled release of BSA in SIF by mixing ALG with CH. Rifampicin is characterized by poor water solubility and therefore low bioavailability after oral administration, which combined with daily admission for at least 4 months, could carry a risk of treatment failure. Lacerda L. et al. [199] designed ALG microparticles with rifampicin enriched with CH to prolong drug release and demonstrated rifampicin release at pH 1.2 not exceeding 20% and 100% release in 250 min at pH 6.8. Wang S. et al. [200] designed ALG–CH films with ethyl cellulose to improve the bioavailability of zolmitriptan and etodolac after buccal administration by reducing the first-pass effect and by decreasing the risk of degradation of these drugs at acidic gastric pH. The films were manufactured from a mixture of ALG and CH to improve the mechanical properties and bioadhesion of the films to the buccal mucosa. Mechanical properties are of significant importance with regard to the films, as higher values of folding resistance or tensile strength provide greater durability and strength of the films during application. Thus, buccal films composed of ALG and CH seem to be a better option in this regard, as they have higher values of the aforementioned parameters, compared to the films composed of pure ALG or CH. A formulation composed of ALG and CH in equal parts proved to be the most mechanically robust, which is presumably due to the highest number of amide bonds formed between the carboxyl groups of ALG and the amine groups of CH. Increases in bioadhesion strength and time, polymer film thickness, and tensile strength were also observed with an increase in the molecular weight of the CH used in the formulation, which is likely related to the greater number of interactions occurring between larger molecules. The enrichment of the ALG films with CH also affected the release profiles of the zolmitriptan and etodolac. An increase in the total amount of drug released for both substances at pH 8 was observed for the compounded formulations than from the single ones. Moreover, the ALG–CH films possessed a more prolonged and controlled release profile than the ALG films; thus, it could be assumed that such a CH-enriched form of the drug reduces the burst effect significantly. Abruzzo A. et al. [201] proposed ALG–CH inserts fabricated by freeze-drying as a solid form of vaginal drug containing chlorhexidine digluconate, a substance with potent antimicrobial activity and a broad spectrum of action, in order to increase the effectiveness and reduce the treatment time of vaginal infections, one of the most common gynecological disorders. ALG was selected to prepare the drug form, due to its mucoadhesive properties, and CH, to enhance mucoadhesion and allow for control of drug release. Inserts were designed to reduce the inconveniences of vaginal administration, such as the short residence time, due to the cleansing mechanisms of the vaginal mucosa and the associated leakage of residues of the applied form of the drug, which contaminate undergarments. Sodium ALG and CH were dissolved separately in acetic acid solution, and then, appropriate amounts of CH were added to the ALG solution to obtain formulations with specific ALG–CH ratios: 90:10, 70:30, 50:50, 30:70, 10:90, which were then centrifuged and washed. At vaginal fluid pH 4.5, CH shows higher mucoadhesion than ALG, which is related to the interaction between amino groups of CH with negatively charged sialic acid residues of mucin, which was also observed in the study. Formulations with higher CH content possessed higher mucoadhesion compared to those with pure ALG. A release study conducted in phosphate buffer at pH 4.5 showed greater release control for the ALG–CH formulation compared to the ALG formulation, and thus, the blended formulations provided longer drug action at the site of administration, which might translate favorably into treatment efficacy. Moreover, the least amount of water uptake was observed for the formulation containing ALG–CH in equal parts, which might reduce the discomfort associated with this form of administration, caused by painful mucosal water draining. A study of antibacterial activity showed that drug-free inserts exhibited a weak antibacterial effect derived from the properties of CH, which can further be utilized to complement the drug effect in the treatment of vaginal infections.

### 6.3. ALG Blends with Gelatin

**Figure 8:**
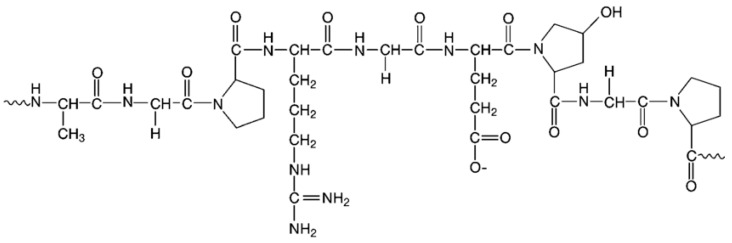
GEL structure.

Gelatin (GEL) is a natural polymer with peptide structure, obtained in the process of collagen breakdown, which is a component of animal connective tissue, bones, and skin coatings. GEL is composed of the amino acids, to the greatest extent, glycine, proline, hydroxyproline, and glutamic acid (25.5%, 18%, 14.1%, 11.4%, respectively), and to a lesser extent, alanine, arginine, asparagine and others (Figure 8) [202]. In addition, there are peptide sequences in the GEL structure that are sensitive to metalloproteinases (MMPs), allowing it to be degraded by cells [203,204].

There are two types of GEL, type A and type B, depending on the pH of the polymer extraction environment. An acidic environment, such as hydrogen chloride, dilute sulfuric acid, can obtain type A GEL. An alkaline environment, such as calcium hydroxide or sodium hydroxide, provides type B GEL. GEL does not dissolve in cold water, while it is well soluble in hot water. It is recommended to swell in cold water before dissolving [205]. GEL is characterized by its ability to form a gel, the structure of which can be enhanced by crosslinking with microwaves [206], formaldehyde [202], glutaraldehyde [207], or carbodiimide [208]. Due to its biocompatibility, biodegradability, gelling ability, natural origin, edibility, and water solubility, GEL is widely used in the pharmaceutical, food, and cosmetic industries as a gelling, stabilizing, and thickening agent. GEL is widely utilized in pharmaceutical technology in the production of hard and soft capsules, hemostatic dressings, and suppositories and as an emulsifier, stabilizer or binding agent [209]. ALG possess many advantages as materials used in microparticle technology; however, they provide poor attachment to encapsulated cells. Therefore, GEL is included into the formulation. This polymer introduces the so-called RGD sequence (containing the amino acids arginine, glycine, asparagine sequentially), which provides anchor points to the cells, thereby increasing their attachment. This approach was applied to the design of ALG–GEL microspheres containing human adipose tissue stem cells (hADSCs) employed in tissue engineering. The ALG formulation was enriched with GEL to obtain a platform that resembles the structure of the extracellular matrix as much as possible, thus improving cell attachment and increasing cell proliferation and differentiation toward adipocytes [210]. SA and B-type GEL were utilized to produce microspheres. In the study, increased proliferation of hADSC cells was observed for the blended formulations, compared to the formulation composed of pure ALG. Moreover, the increase in proliferation was directly proportional to the GEL content, which presumably might be related to the increased anchor points provided by GEL and to the increased delivery of nutrients to the cells through the increased pores of the matrix that resulted from the reduced concentration of ALG in the formulation [211]. Higher differentiation of hADSC cells into adipocytes was also observed in the blended formulations than in pure ALG. Morshedloo F. et al. [212] designed an ALG–GEL injectable subcutaneous hydrogel containing encapsulated chondrocytes to accelerate soft tissue regeneration while minimizing interference with the tissue continuity, as provided by this drug formulation. SA modified with the addition of a phenolic group to enable enzymatic crosslinking, porcine GEL type A and human C28/12 chondrocytes were utilized to prepare formulations. Obtained results confirmed the validity of using an ALG–GEL blend as an injectable hydrogel, as the gelation time of such a mixture increased from 2.9 min for a formulation containing only ALG, to 5.1 min for a blended formulation, preventing premature gelling of the structure. In addition, the blended formulation was characterized by accelerated and increased swelling, compared to pure ALG, which might probably be related to the hydrophilic nature of GEL, important in the context of supplying nutrients to the cells in the produced hydrogel. Mechanical properties of an injectable hydrogel are crucial and depend strictly on the site of injection, and thus, their values need to be adjusted depending on the intended use of the drug and the type of injury. They showed a 35% reduction in the mechanical strength of the hydrogel after the addition of GEL, indicating that the designed hydrogel will be able to find application in the treatment of soft tissue disorders. Similar to the previous study, an increase in cell proliferation was demonstrated when the formulation was enriched with GEL. Sarker B. et al. [213] enriched ALG microcapsules by adding type A porcine GEL to increase the degradability of the formulation during tissue regeneration. All of the composite formulations showed an increase in degradability compared to the ALG formulation and an increase in the swelling index. Sarker B. et al. [214] also fabricated films consisting of dialdehyde-crosslinked SA to improve the degradability of the formulations as well as type A porcine GEL to deliver the RGD sequence to improve the adhesion of encapsulated cells, normal human dermal fibroblasts (NHDF), which are among the primary cells involved in wound healing, angiogenesis, and regeneration. The study showed improved adhesion, proliferation and survival of NHDF cells in blended formulations, compared to ALG formulations, and with the increase in the GEL content in the formulation, the rate and degree of degradation of the films increased. Leone G. et al. [215] designed ALG–GEL matrix tablets to prolong the release of red yeast rice extract (RYR) containing lovastatin. Lovastatin administered orally in the form of RYR is more easily absorbed than the pure substance and has fewer side effects, such as muscle pain [216]; thus, it could be an alternative to traditional therapies. In order to prolong the release of RYR from the drug formulation for up to 7-8 h, and to ensure constant levels of the drug in the body after an overnight dose, the ALG formulation was enriched with GEL. The study yielded a formulation of 60% GEL and 40% ALG with the most favorable properties, as about 50% of the lovastatin dose was released in about 7 h, which is in accordance with the daily cycle of cholesterol biosynthesis. In a release study conducted at pH 7.4, a significant reduction in drug release was observed from the compounded matrices compared to bare RYR; moreover, the formulations with the lowest GEL content (GEL10:ALG90, GEL20:ALG80) had a release profile similar to pure RYR; thus, they did not modify release, presumably because the relatively high ALG content caused accelerated disintegration of the formulation. There was no linear relationship between the content of both polymers in the formulation and the release profile, as the GEL90:ALG10, GEL30:ALG70, and GEL40:ALG60 formulations showed too prolonged release (about 20%, 30%, and 40% in 5 h, respectively), while the GEL80:ALG20, GEL70:ALG30, GEL60:ALG40, and GEL50:ALG50 formulations showed the most favorable extended-release profiles (about 100% release in 24 h). These formulations showed a lower elastic modulus than GEL90:ALG10, GEL30:ALG70, and GEL40:ALG60, resulting in a lower release retardation, with the entire dose of the drug being released within 24 h. The GEL60:ALG40 formulation also showed the greatest HMG-CoA reductase inhibitory activity, while all formulations had a slightly stronger inhibition of cholesterol synthesis compared to bare RYR. When compared to synthetic lovastatin, the reduction appeared more pronounced.

GEL is also combined with ALG in the formulation of wound dressings, due to its hemostatic properties and the previously mentioned RGD sequence, which provides anchor points to cells. Afjoul H. et al. [217] proposed an ALG–GEL scaffold using the freeze-gelation method, designed to be applied as a wound dressing for wounds for which a skin graft was already needed. SA- and A-type GEL were employed to prepare the formulations. The concentration of ALG in all formulations was equal (3%), while the concentration of GEL varied from 1%, 2% to 3% (samples ALG:GEL1, ALG:GEL2, ALG:GEL3). The scaffold placed in the wound should be characterized by mechanical properties that can endure application to the site of injury, with the tensile strength adequate to the wound healing process (1-20 MPa [218,219]). A significant increase in the tensile strength of the scaffold was observed when the concentration of GEL in the formulation was doubled (from ALG:GEL1 to ALG:GEL2), but no statistically significant differences were noted between the ALG:GEL2, and ALG:GEL3 formulations. Thus, in this case, the improvement in the mechanical properties of scaffolds with an increase in the ratio of GEL in the formulation is noticeable, but only up to a certain point, and more research and sampling are needed. Degradability testing showed increased degradation of the scaffold with increasing GEL content in the formulation, which is desirable for soft tissue regeneration. For regeneration of harder tissue, such as bone, the value of this parameter would need to be improved, so that the scaffold stays longer at the application site, as bone healing is slower. Cell adhesion studies of the mouse fibroblasts L929 showed an increase in cell adhesion with an increase in the GEL content of the formulation. Ture H. [220] proposed an ALG–GEL dressing enriched with the addition of hydroxyapatite, a biocompatible, non-toxic and non-immunogenic substance, to increase mechanical strength. In the study, SA, bovine GEL type B, hydroxyapatite, and tetracycline hydrochloride as the model drug were employed, and the formulations were carried out with ratios of ALG to GEL of 60:40, 50:50, and 40:60. In accordance with the results of other authors [212,213], swelling was reported to increase with the increase in GEL content in the formulation; moreover, the increase in GEL content caused an increase in drug release, while the addition of hydroxyapatite, by crosslinking the polymer structure and by increasing the mechanical strength of the dressing, caused a delay in drug release. The aforementioned studies indicated that by modulating GEL content in ALG formulations, values such as cell adhesion or degradability, as well as the swelling index, and drug release profile, could be adjusted to design a drug formulation with the desired properties.

### 6.4. ALG Blends with Carrageenans

**Figure 9:**
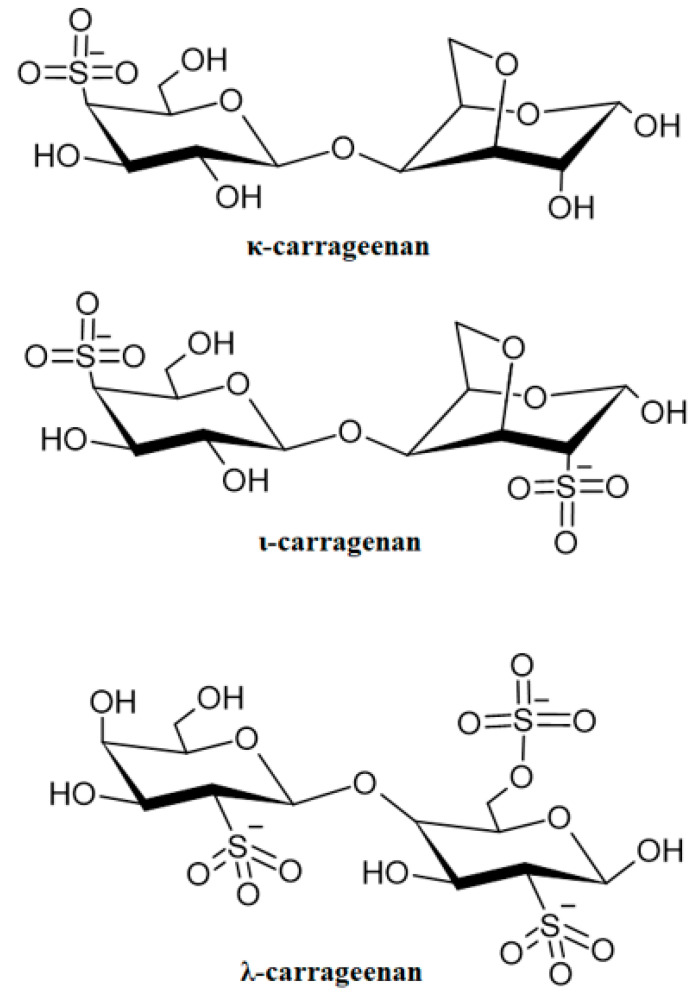
Chemical structure of CAR.

Carrageenans (CAR) are a group of polysaccharides obtained by extraction from the red seaweed *Rhodophyta*, most frequently from the species *Chondrus*, *Euchema*, *Gigartina* and *Hypnea* [221]. In their structure, galactose and anhydrogalactose connected by glycosidic bonds as well as sulfonic moieties can be distinguished, as characteristic for the polymer substituents in the chain. CAR occur as potassium, sodium, calcium, magnesium or ammonium salts. Depending on the content of sulfonic groups, and the presence of anhydrogalactose, several varieties of CAR with different gelling abilities are diversified: kappa (κ), iota (ι), and lambda (λ) (Figure 9) [221,222]. The gelation capacity of CAR depends on the content of anhydrogalactose in the chain, λ-CAR does not contain it at all, and thus, its gelation capacity is negligible. ι-CAR contains about 30% anhydrogalactose in the chain; therefore, it shows higher gelation capacity. Meanwhile, κ-CAR contains the highest content of this sugar in the chain; thus, it has the highest gelation capacity. Nevertheless, all CAR are mucoadhesive polymers. CAR are characterized by a variety of applications in medicine and pharmaceutical technology, many of which are under research. These polymers might be utilized in drug formulation technology as components of tablet matrices, stabilizing and binding agents, and disintegrating, solubilizing substances, and thickening agents. They can equally be applied as coating substances, or release modifiers, since they have gelling ability. κ-CAR is also applicable in pellet technology, which compared to conventional pellets manufactured from microcrystalline cellulose, are characterized by faster disintegration, resulting in faster drug release. In addition, due to the structural similarity of CAR to glycosaminoglycans, an essential component of the extracellular matrix, they can be applied in tissue engineering and bone and cartilage regeneration. When applied as an ingredient in theophylline tablets, ι-CAR provided prolonged theophylline release up to 8–12 h [223]. κ-CAR was utilized to formulate acyclovir vaginal tablets [224] and tenofovir globules [225] in order to achieve controlled drug release. In addition, due to their ease of storage and stability, CAR films can be a form of dressing that when enriched with other polymers, provides release modifications. CAR are also being investigated for employment in extended-release oral suspension technology, for example, with ambroxol, which provided greater control of drug release compared to suspension without CAR [226]. Due to its antiviral properties, CAR are being investigated for use in intranasal administration, such as intranasal inserts with gold nanoparticles [227]. Moreover, CAR were used to fabricate microcapsules containing proteins [228], as well as probiotic bacteria [229], microspheres, beads [230,231], and nanoparticles [232]. Numerous studies have demonstrated antiviral properties of CAR against *Herpes* [233], *Cytomegalovirus, Human Papillomavirus* [234], and *HIV* [235] viruses. CAR have also been used to induce inflammation in animals while testing the effects of anti-inflammatory drugs [221].

Combinations of ALG with CAR are less common than the aforementioned mixtures with PEC, CH, or GEL; however, some effects of CAR on the properties of the ALG-based drug formulations might be observed. Magnetic pH-sensitive ALG–CAR particles designed by Mahdavinia G.R. et al. [236] showed that κ-CAR affected, inter alia, swelling properties and the release profile of this drug formulation. SA, κ-CAR at various concentrations, and riboflavin as a release test substance were utilized to fabricate formulations, in order to observe how CAR affects the swelling of the drug form, and the release profile. With increased CAR content in the formulation, there was an increase in the swelling index, which was presumably related to the increased water absorption, and the fact that the sulfonic groups included in the CAR chain possessed a greater ability to dissociate in an aqueous environment than the carboxyl groups in the ALG chain. The interactions of potassium ions with CAR molecules were weaker than the interactions of calcium ions with ALG; thus, the resulting CAR hydrogel possessed lower mechanical strength than ALG hydrogel, and could absorb more water and swell more. Expanding further on this thought, the swelling of the formulation also depends on the presence of ions in the environment. In the presence of potassium ions, the swelling increases as the CAR content in the formulation decreases, because CAR undergoes gelation in the presence of these ions. In the environment of calcium ions, the opposite trend might be observed, that is, an increase in swelling with an increase in the content of CAR in the formulation, because these ions cause gelation of ALG. The addition of CAR might contribute to the relaxation of the gel structure and thus an increase in swelling. Moreover, higher swelling was also observed for all formulations at pH 7.4 (phosphate-buffered saline, PBS) than at pH 1.2 (hydrochloric acid buffered solution, HBS). This difference became less pronounced as the concentration of CAR in the formulation increased, indicating that formulations with higher CAR content were less pH-sensitive, as indicated by the pKa values of the CAR and ALG functional groups. The results of the swelling study were covered by the results of the release study, as higher release was observed for formulations with higher CAR content, due to the fact that it formed a mechanically weaker gel. Thus, CAR seems to be suitable for drug formulations for not prolonged, or not delayed release. A similar swelling trend as in the previously cited paper [236] was observed when studying ALG–CAR dressings containing biosurfactants [237]. The aim of combining SA with κ-CAR was to achieve an improvement in the dressing’s absorbency and mechanical properties, while adding biosurfactants (rhamnolipids and sophorolipids) to provide the dressing with anti-inflammatory and antimicrobial properties. The swelling study concluded that the combination of these two polymers caused an increase in swelling, compared to formulations consisting of single polymers, without indicating a clear, transparent trend in the relationship between CAR content in the formulation and the swelling process. The pore size of freeze-dried dressings affected the ability to absorb exudate. The greater the porosity (the greater the number of smaller pores), the better that the dressing could handle exudate. The addition of CAR increased the pore size of the dressing. On the other hand, Zia T. et al. [238] observed an improvement in tensile strength and Young’s modulus with increasing κ-CAR content in ALG–CAR dressings enriched by silver with antibacterial properties. In addition, an improvement in wound healing was observed with an increase in CAR content in the formulation after the dressing was applied to the rat wound; thus, increasing the concentration of CAR might lead to faster wound healing. ALG–CAR blends were also being studied for use to encapsulate microorganisms to increase their survival rate and to protect them from the acidic environment in the stomach. Silva Batalha L. et al. [239] encapsulated UFV-AREG1 bacteriophages in an ALG–CAR matrix to increase their stability and to prolong their release. ALG–CH, ALG–CAR, ALG–whey protein, and pure ALG matrices were compared. In terms of bacteriophage protection against the damaging effects of hydrochloric acid, CH combined with ALG showed the weakest protection, whey protein with ALG showed the best, while the ALG–CAR blend provided a medium-level protection against an acidic environment. A release test in simulated intestinal fluid (SIF) after immersion in simulated gastric fluid (SGF) showed the fastest release of microorganisms from the drug formulation in the case of the ALG–CAR formulation. If the objective is merely to achieve protection against acidic pH, then CAR in combination with ALG fulfills this function; however, the prolongation of release is missing in this case. The bacteriophages had the best stability during wet storage for 5 months for the ALG–CAR formulation. Qi X. et al. [240] performed a similar study and compared the effects of adding κ-CAR and LM PEC to SA on the survival of *Lactobacillus rhamnosus GG* (LGG) in a simulated gastric fluid environment. SA by itself at concentrations >2% possessed properties protecting bacteria in an acidic environment. However, high concentrations were accompanied by difficulties in maintaining the spherical shape of the particles. In order to preserve the globular shape of the particles and to simultaneously protect their containment from acidic pH, ALG-based formulations with lower concentrations were enriched with other polymers. The researchers showed that the addition of LM PEC reduced the shrinking of the drug formulation to a greater extent in an acidic environment compared to CAR. During the transfer of particles into simulated intestinal fluid (SIF), an increase in the swelling index for formulations containing LM PEC was observed, while for formulations with CAR, the swelling index decreased significantly, which translated unfavorably into the drug release process. Comparing the cell death rate for the different formulations, the highest values of this index were reported for ALG–CAR formulations, which might be related to the low strength of the gel formed, the increased penetration of acid into the particles during the residence of the drug formulation in the stomach, and damage to the bacterial cells. Thus, this study indicated the need for further research in developing a matrix formulation for encapsulation of bacterial cells. Kim M.H. et al. [241] combined SA with κ-CAR in a bio ink formulation for 3D printing to obtain constructs capable of maintaining their shape, as 3D constructs prepared on an ALG matrix tend to lose their original form. An increase in viscosity and shear modulus as the concentration of CAR in the formulation increased was observed. The blended formulations possessed better shape fidelity than the ALG formulation; thus, the enrichment of ALG with the addition of κ-CAR could be an interesting approach in the technology of ALG 3D constructs. There were also attempts to prolong release from the drug formulation by enriching the formulation with κ-CAR. Zhang Y. et al. [242] developed ALG–CAR beads containing *Brucea javanica* oil with anti-cancer properties in gastrointestinal tumors. Currently available forms of the drug with this ingredient, soft capsules, and injectable emulsions are characterized by very rapid release, low bioavailability and poor thermodynamic stability. A release study conducted at pH 1.2 showed a prolongation of release up to 6 h for blended formulations, compared to pure ALG, as well as an increase in the amount of drug released from 30% for pure ALG to 90% for blended formulations, with no linear relationship between the CAR content of the formulation, and the release profile.

**Table 4:** ALG blends.

ALG Blends	Drug Form	Effect	Reference
ALG–PEC	Microcapsules Films Particles	Increased encapsulation efficiency Loss of spherical shape Improved drug retention Release prolongation Increased elongation at break Maintaining shape after rehydration Increased tensile strength Decreased gel strength Increased swelling Increased encapsulation efficiency Increased mucoadhesion Release prolongation	[176,177,178] [176,177,178] [176,178] [177,178] [182,184] [182] [184] [183] [183] [183,185,186] [185,186] [185,186]
ALG–CH	Dressings Bone defect filling Particles Films Inserts	Increased antibacterial effect Increased anti-inflammatory effect Reduced brittleness Increased Young modulus Release prolongation Increased folding resistance and tensile strength Increased bioadhesion strength and time Release prolongation Increased mucoadhesion Release prolongation	[195] [195,196] [197] [197] [198,199] [200] [200] [200] [201] [201]
ALG–GEL	Microspheres Injectable subcutaneous hydrogel Microcapsules Films Matrix tablets Dressing	Increased proliferation and differentiation Gelation time prolongation Increased swelling Increased degradability Improved cell adhesion, proliferation and survival Release reduction Mechanical properties improvement Increased degradability, cell adhesion Increased swelling	[210] [212] [212] [213] [214] [215] [217,220] [217] [220]
ALG–CAR	Particles Dressings Microcapsules Bio ink for 3D printing	Increased swelling Increased release Release prolongation Increased swelling Increased pore size Increased tensile strength and Young’s modulus Improvement in wound healing Protection against acidic pH Shape fidelity Increased shear modulus	[236] [236,242] [242] [237] [237] [238] [238] [239] [241] [241]

## 7. Conclusions

Due to numerous beneficial properties, ALG are highly versatile polymers. However, disadvantages such as difficulty providing modified drug release, poor mechanical properties, fragility, low flexibility, or poor cell adhesion might limit their pharmaceutical applications. To overcome these shortcomings, blends of ALG with various natural or synthetic polymers are utilized. It was shown that ALG–PEC combinations possessed increased encapsulation efficiency [176,177,183] and prolonged drug release compared to pure ALG formulations [184,185,186], which enabled a reduction in the frequency of dose administration. An increase in gel strength, tensile strength, and elongation at break in films improved the application properties of ALG–PEC dressings and helped to maintain shape while absorbing wound exudate, in contrast to pure ALG dressing [182]. CH provides antimicrobial properties and is beneficial to the wound healing process through modulation of the inflammatory processes, stimulation of fibroblast growth, and collagen synthesis [197]. Enrichment of ALG formulations with CH might result in an improvement in the mechanical properties and prolongation of the drug formulation residence time at the application site in bone regeneration. In order to improve poor cell adhesion to ALG matrices, combinations of ALG with GEL might be utilized, due to the RGD sequence provided by GEL [210,214,217], which also enhanced proliferation and differentiation of encapsulated cells [210,212,214]. Furthermore, the addition of GEL increased swelling [212,220] and thereby facilitated cellular access to nutrients and accelerated matrix degradation in the tissue [213,217]. From the presented studies of ALG blends with CAR, the results are divergent. Enrichment of the ALG formulations with CAR most frequently resulted in swelling increases [236,237], although a decrease was also demonstrated [240]. Reduction of the absorption capacity with the addition of CAR was noted [237], however, an improvement in the healing process was reported [238]. CAR was also considered in the use in encapsulating probiotic bacteria and bacteriophages [239,240] to increase the stability and protection of microorganisms. CAR might turn out to be a promising additive in 3D construct technology, as it significantly improved shape fidelity compared to pure ALG [241].

## Figures and Tables

**Table 1:** ALG properties and applications [7,8,9,10,11].

ALG Derivative	Physical Form	Water Solubility	Pharmaceutical Applications
Alginic acid	Odorless and tasteless, white to yellowish fibrous powder	Swells in water without dissolving	Binder and disintegrating agent (1–5%) in tablet technology; thickening and suspending agent in semi-solid drug forms
Sodium alginate	Odorless, tasteless, white to pale yellow-brown powder	Good water solubility with forming viscous solutions	Binder and disintegration agent in tablet technology in sustained-release dosage forms; thickening and suspending agent in o/w emulsions, creams, pastes; in microparticles and nanoparticles technology; mucoadhesive dosage forms
Calcium alginate	Odorless, tasteless, white to pale yellow-brown powder or fibers	Practically insoluble in water	Tablet disintegrant, wound dressings, floating dosage systems, dosage forms with modified release
Ammonium alginate	White to yellow-brown threaded, granular or powdered forms	Slowly dissolves in water	Emulsifying agent, film-former, humectant
Propylene glycol alginate	Practically odorless and tasteless white to yellowish granular or fibrous powder	Soluble in water mixed with ethanol 95% up to 60% (*w*/*w*) (depending on esterification degree)	Emulsifying, stabilizing, gelling and suspending agent

**Table 3:** ALG Pharmaceutical applications.

ALG Pharmaceutical Applications	Reference
Semi-solid dosage forms	[1,8]
Microparticles	[91,92,93,94,95,96,97,98]
Mucoadhesive dosage forms	[90,102]
Nanoparticles	[109,110,111,112]
Tablets technology	[113,114,115]
Tissue engineering	[118,130]
Bone and cartilage regeneration	[120,121]
Wound dressing	[138,139]
Cell culture	[124,125,126,127,128,129]

## Data Availability

Not applicable.
